# Galcanezumab-Induced Myasthenia Gravis-Like Symptoms

**DOI:** 10.7759/cureus.40127

**Published:** 2023-06-08

**Authors:** Adnan A Mubaraki

**Affiliations:** 1 Department of Medicine, Taif University, Taif, SAU

**Keywords:** vision changes, neuromuscular diseases, calcitonin gene-related peptide (cgrp), migraine disorder, ocular-myasthenia-gravis

## Abstract

Fluctuating weakness affecting the ocular, bulbar, and/or appendicular muscles is characteristic of myasthenia gravis. Autoimmune components and certain drugs have been implicated in the pathophysiology of this disease. I report a case of chronic migraine in which the patient developed symptoms of myasthenia gravis after using galcanezumab, the recently approved anti-calcitonin gene-related peptide (anti-CGRP). This case shows that anti-CGRP medications could affect the neuromuscular junction and cause such symptoms. Moreover, this case illustrates the clinical approach and management of such a presentation.

## Introduction

Myasthenia gravis is an autoimmune neuromuscular junction disorder that causes fatigue and fluctuating weakness, and it can affect any striated muscle [1]. Clinically, it can vary from pure ocular weakness to generalized weakness, including the bulbar and respiratory muscles [1]. Several monoclonal antibodies targeting calcitonin gene-related peptides (CGRP) have been recently approved to treat episodic and chronic migraine [2]. In this study, I report a case of seronegative ocular myasthenia gravis, which developed after using galcanezumab for migraine.

## Case presentation

A 42-year-old man has suffered from migraines for the past six years. He tried various prophylactic medications, including propranolol and amitriptyline. His migraine attacks did not significantly improve. I discussed with him the newly approved monoclonal antibodies. It was decided to start him on galcanezumab. Two months later, his headaches improved in frequency and intensity. But he reported that while working on a patient as a dentist, he developed double vision, which impaired his ability to continue the procedure. He thought that this issue was due to overwork. His condition improved after one hour. The next day, while again at work, he noted the same symptom, but this time it was associated with neck pain and heaviness. He took time off from work, hoping his symptoms would improve. However, he started to get more fatigued. These symptoms were more pronounced in the afternoon. While looking in the mirror, he noted mild left eyelid ptosis. He did not have any history of swallowing difficulty or arm or leg weakness. He stated that sensory symptoms did not affect his extremities. Bladder and bowel functions were preserved.

Examination showed subtle, fatigable ptosis, more affecting the right eye, and horizontal diplopia upon prolonged right gaze. Manual motor testing of both upper and lower extremities was unremarkable. Sensory evaluation was inconclusive as well.

Because his symptoms were intermittent and more pronounced by the end of the day, myasthenia gravis was suspected. The possibility of galcanezumab-induced myasthenia gravis was increased owing to the onset of these symptoms after using this medication. Clinically, he improved after stopping galcanezumab and taking acetylcholinesterase inhibitors for a short period of time. Edrophonium and ice pack tests were not done. These tests have been described as bedside tests to evaluate the temporary improvement of clinical symptoms of myasthenia gravis after using edrophonium, which is considered a short-acting acetylcholinesterase inhibitor, or an ice-pack test over the eyelids of those patients and monitor for improvement of ptosis afterward.

**Figure 1:**
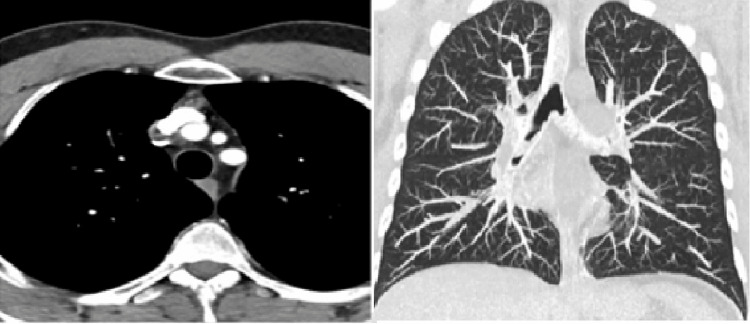
CT-chest with contrast shows normal lung volume and thymus gland.

Both acetylcholine receptor antibodies and anti-muscle-specific kinase (MuSK) antibodies were negative. The thyroid function test was normal. A computed tomography (CT) scan of the chest with contrast did not show thymus abnormalities (Figure 1). The patient refused to do nerve conduction and repetitive nerve stimulation tests.

Galcanezumab treatment was stopped. Because his symptoms were only ocular and interfering with his work as a dentist, he was started on pyridostigmine 60 mg twice a day. One month later, he was seen for a follow-up. Minimal diplopia was present, but only after overwork. The dose of pyridostigmine was increased to three times a day.

Two months later, he reported no further symptoms. I advised him to continue this regimen for another three months. The pyridostigmine treatment was then stopped. Upon follow-up two months later, the patient was stable.

## Discussion

Galcanezumab, fremanezumab, and erenumab are monoclonal antibodies that were US-FDA-approved in 2018 to treat episodic and chronic migraine by targeting CGRP [3]. CGRP is a neurotransmitter that is important in the migraine cascade and contributes to various physiological functions in the body [3,4]. CGRP is also an important regulatory protein in neuromuscular junction function and muscle contraction owing to its involvement in acetylcholine receptor and acetylcholinesterase regulation [5]. This patient used this medication for two months before observing the abovementioned symptoms, which suggests that this drug has cumulative side effects. Causality can be suggested based on the onset of these symptoms after using galcanezumab, the presence of negative antibodies against the acetylcholine receptor and MuSK, and clinical improvement after stopping galcanezumab treatment. Upon reviewing the literature, a similar case has been previously reported, but after using erenumab, which also improved after stopping it [6]. Both galcanezumab and erenumab exert their action by blocking the CGRP by either blocking its ligand or receptor [7]. Through this mechanism of action, the possibility of triggering myasthenia-like symptoms or worsening the symptoms of myasthenia gravis patients is considered but not confirmed. Additional studies are needed to confirm or dispute this hypothesis.

## Conclusions

In conclusion, recently approved monoclonal antibodies have proven effective in treating migraine. The novel mechanism of action targeting CGRP throughout the body may have different side effects. Thus, additional studies are needed to evaluate the long-term use of these medications and their associated side effects. To the best of my knowledge, this is the first reported case of myasthenia-like symptoms after using galcanezumab.
